# Targeting Local Recurrence After Surgery With MRI Imaging for Prostate Cancer in the Setting of Salvage Radiation Therapy

**DOI:** 10.3389/fonc.2022.775387

**Published:** 2022-02-15

**Authors:** Raphaële Renard-Penna, Jules Zhang-Yin, Sarah Montagne, Laurene Aupin, Eric Bruguière, Mouna Labidi, Igor Latorzeff, Christophe Hennequin

**Affiliations:** ^1^Academic Department of Radiology, Hôpital Pitié-Salpétrière, Assistance Publique des Hôpitaux de Paris, Paris, France; ^2^Sorbonne University, Paris, France; ^3^Nuclear Medicine Department, Tenon Hospital, Assistance Publique des Hôpitaux de Paris (APHP), Paris, France; ^4^Department of Imaging, Clinique Pasteur, Toulouse, France; ^5^Department of Oncology, Saint-Louis Hospital, Université de Paris, Assistance Publique des Hôpitaux de Paris, Paris, France; ^6^Department of Radiotherapy, Clinique Pasteur, Toulouse, France

**Keywords:** prostate cancer, prostatectomy, MRI, diffusion magnetic resonance imaging, local neoplasm recurrence, radiation therapy

## Abstract

Magnetic resonance imaging (MRI) is being increasingly used for imaging suspected recurrence in prostate cancer therapy. Functional MRI with diffusion and perfusion imaging has the potential to demonstrate local recurrence even at low PSA value. Detection of recurrence can modify the management of postprostatectomy biochemical recurrence. MRI scan acquired before salvage radiotherapy is useful for the localization of recurrent tumors and also in the delineation of the target volume. The objective of this review is to assess the role and potential impact of MRI in targeting local recurrence after surgery for prostate cancer in the setting of salvage radiation therapy.

## Highlights

- MRI can diagnose local prostate cancer recurrence after radical prostatectomy even at low PSA value.
- DWI-MR is almost comparable with DCE-MRI in detecting local recurrence.
- MRI enables a better delineation of identified recurrence and a precise anatomical analysis of the prostate bed than CT.

## Introduction

Radical prostatectomy (RP) is a standard treatment for localized prostate cancer (PCa). Of 100 men treated with RP, approximatively 15–60 will develop biochemical recurrence (BCR) within 10 years (1, 2). The measurement of prostate-specific antigen (PSA) is a key component in follow-up after RP because of the so-called BCR which precedes clinical recurrence (3, 4). BCR is defined as PSA ≥0.2 ng/ml, followed by a second confirmatory serum PSA measurement of greater than or equal to 0.2 ng/ml (5). Commonly, BCR signed an early recurrent disease and include local recurrence (LR) in the prostate fossa as well as lymph node and/or bone metastases. The pattern of recurrence after RP is predominantly local (≈60%) with a relatively low incidence of metastatic failure (6).

Some clinico-pathological features and characteristics of PSA recurrence represent important variables when trying to distinguish between a local and distant recurrence. Indeed, Gleason score (GS) ≥8, seminal vesicle involvement, or pelvic lymph node (LN) invasion at the time of surgery seem to be correlated with distant recurrences. Similarly, a BCR occurring within 6 months of RP or a PSA doubling time of less than 12 months are strong indicators of metastases (7, 8). On the contrary, PSA increases of more than 3 years post-RP, PSA doubling time greater than 12 months, original Gleason score ≤7, pT3a stage (extracapsular extension without seminal vesicle infiltration), and positive surgical margins suggest that the relapse is more likely to be local (9–12).

The options for treatment of recurrence after RP are, according to the European Association of Urology (EAU), salvage radiotherapy (SRT) at least to the prostatic fossa, continuous or intermittent hormonal therapy (HT), or simple monitoring (5). Proper identification of recurrence location is important for subsequent treatment decisions because, in local recurrence or loco-regional LN metastasis, curative local treatments as SRT can still be feasible and improve disease-free survival and overall survival (8, 13, 14). In the case of distant metastasis, the patient would get most of the time only of palliative treatment (15). Imaging is a staple tool for discovering the site(s) of recurrence and distinguishing between local and distant metastatic disease.

Positron emission tomography (PET) with choline or PSMA has become the reference in detecting metastases in the skeleton, in distant lymph nodes, and in the viscera (16).

Prostate magnetic resonance imaging (MRI) with functional sequences allows early detection of local recurrence and may also be a valuable correlative imaging modality for equivocal PET findings (17). MRI provides a better anatomical delineation of recurrence for the CTV delineation (18), and it allows the delivery of higher radiation doses to a specific recurrence site.

However, the role of MRI in this indication is still discussed and has not been validated (19). The aim of this review is to address the role of MRI for the diagnosis and targeting of local recurrence after surgery in the setting of SRT.

## MRI After RP: The Optimal PSA Cut–off

If the disease recurrence is suspected to be local and/or regional, the most common approach is to irradiate the surgical bed as early as possible with cutoff PSA values for referral of 0.1–0.5 ng/ml usually suggested. It has been shown that the best results of SRT are obtained when PSA values are low (20). However, with those PSA values, the probability of finding exact location of the recurrence is low. In a series reporting a high sensitivity (80%–95%) of MRI, usually the PSA value is over 1 ng/ml or local recurrence is palpable on digital rectal examination (DRE) (21, 22). For lower PSA values, Buergy et al. (23) were able to detect local recurrence at PSA values below 0.5 ng/ml with a minimum level of 0.31 ng/ml but were not able to detect recurrence for PSA level below 0.3 ng/ml. In a cohort of 183 patients with BCR after RP, Dirix et al. (24) found that MRI indicated a suspected macroscopic recurrence for 46 (25%) patients which was local in 22 (49%), mainly in the peri-anastomotic region for 11 patients (6%) and in pelvic lymph nodes in 23 (50%). The mean PSA value was higher for patients with a positive MRI (1.4 ng/ml vs. 0.4 ng/ml; *p* < 0.001), but relapse could be located in 13 (7%) patients with a PSA <0.5 ng/ml. When the PSA value was ≤0.3 ng/ml, Liauw et al. (25) observed a local recurrence on MRI only in 11/88 patients (13%) of the patients. Linder et al. (26) showed in their study of 132 patients with a median PSA of 0.59 ng/ml (range <0.1–13.1) that local recurrence was identified by multiparametric magnetic resonance imaging (mpMRI) in 124 patients (94%) with a median lesion size on MRI of 1 cm. The sensitivity of MRI was 91% with a specificity of 45%; the positive predictive value (PPV) and negative predictive value (NPV) were respectively 85% and 60%. For identifying local recurrence on MRI, the optimal PSA cutoff appears to range from 0.3 to 0.54 ng/ml and the PSA kinetic is also a strong predictor for positive MRI findings even with low PSA values (27).

## MRI After RP: How?

The protocol is the same as that performed for tumor detection, compliant with European Society of Uro-Radiology guidelines with T1-weighted, T2-weighted, diffusion-weighted (DW), and dynamic contrast-enhanced (DCE) imaging sequences (28). T2W imaging is always used for anatomy orientation and evaluation of signal patterns after surgery. Functional imagery including diffusion with high *b*-value and perfusion imaging allow the important differentiation between recurrent cancer, residual prostate tissue, inflammatory tissue, and fibrosis.

## Normal Pelvic Anatomy After Radical Prostatectomy

**Figure 1:**
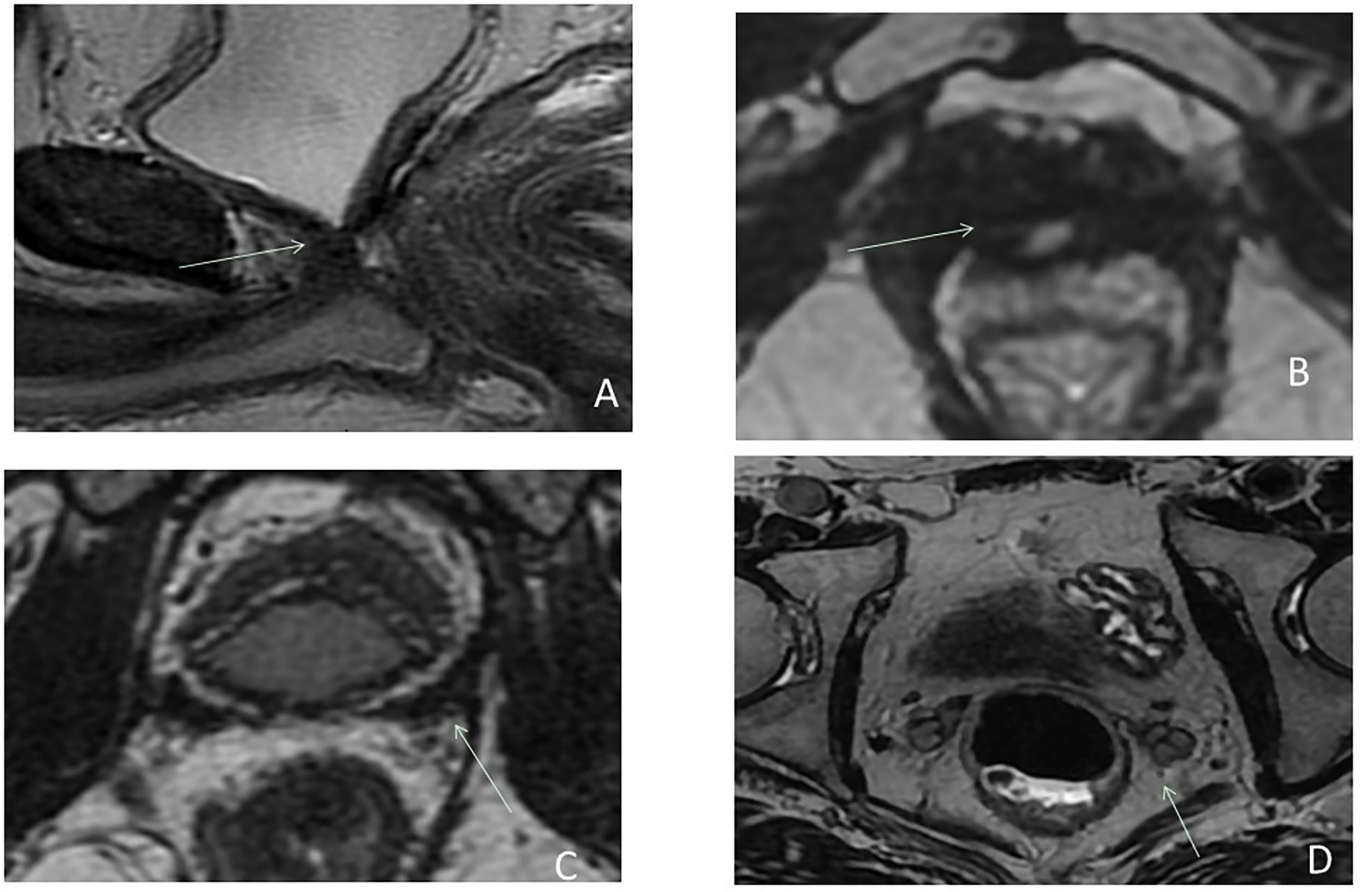
Postsurgical imaging findings after radical prostatectomy on sagittal **(A)** and axial **(B–D)** T2-weighted imaging: bladder neck descended into the prostatectomy fossa with a more conical shape **(A)**; the VUA anastomosis (arrow) **(A, B)** visualized as a ring of postoperative fibrosis on low signal intensity inferior to the bladder neck. Seminal vesicles which are supposed to be removed in a classical RP demonstrate a low signal intensity **(C)** (postoperative fibrosis), but may be retained in part and can be seen in their presurgical locations with their characteristic tubular structure on T2W **(D)**.

RP includes total removal of the prostate and seminal vesicles, along with pelvic lymph node dissection. Postradical prostatectomy MRI findings include descent of the bladder which is anastomosed to extra prostatic distal urethra. The vesico-urethral-anastomosis (VUA) should be visualized as a ring of postoperative fibrosis with low signal intensity on all sequences (**Figure 1**). Seminal vesicle (SV) which are supposed to be removed, may be retained in part with postoperative findings highly variable: 20% of the patients had SV remnants, with similar location of the preoperative SV position, with an additional 38% with fibrotic SV tips (21) (**Figure 1**).

## Location of Recurrence

The most common site of local recurrence after RP is the VUA, but recurrent masses can occur anywhere in the prostatectomy bed, including the retrovesical space, the bladder neck, near the seminal vesicles bed, or adjacent to the vas deferens (27, 29). In a series of 114 patients with a biological relapse after RP, local recurrence was seen on ultrasound at the anastomotic site (66%), the bladder neck (16%), and posterior to the trigone (13%) (30). MRI could help to define more accurately the target volume and so decrease toxicity (31). Moreover, the identification of a local recurrence is predictive of a better efficiency of SRT (32). The main series of local recurrence detected in MRI are presented in **Table 1**.

**Table 1:** Locations by MRI of local recurrences after radical prostatectomy.

Author	No. of patients	Positive MRI (%)	Location of the local relapse: No. of patients (%)				
			Peri-anastomotic	Retrovesical	Bladder neck	Seminal vesicles	Lateral/anterior
Connolly et al. (30)	114	61 (54)	40 (66)	8 (13)	10 (16)	–	–
Sella et al. (22)	41^a^	39 (95)	12 (29)	17 (40)	–	9 (22)	4 (9)
Liauw et al. (25)	88	21 (24)	14 (67)	7 (33)	–	–	
Panebianco et al. (33)	242	226 (93)	61 (27)	41 (20)	37 (16)	31 (14)	53 (23)
Park^b^ et al. (34)		113	93 (79)	7 (6)	18 (15)	–	–
Hernandez^c^ et al. (27)	70	33 (47)	19 (27)	4 (6)	1 (1.5)	2 (3)	
Dirix et al. (24)	183	46 (25)	11 (50)	2 (9)	–	9 (41)	–
Breen et al. (32)	386	216 (56)	127 (59)	28 (13)	61 (28)	–	–

a Localized relapse confirmed.

b Only positive MRI were described.

c Penile bulb: one patient.

## Presentation of Recurrence on MRI

**Figure 2:**
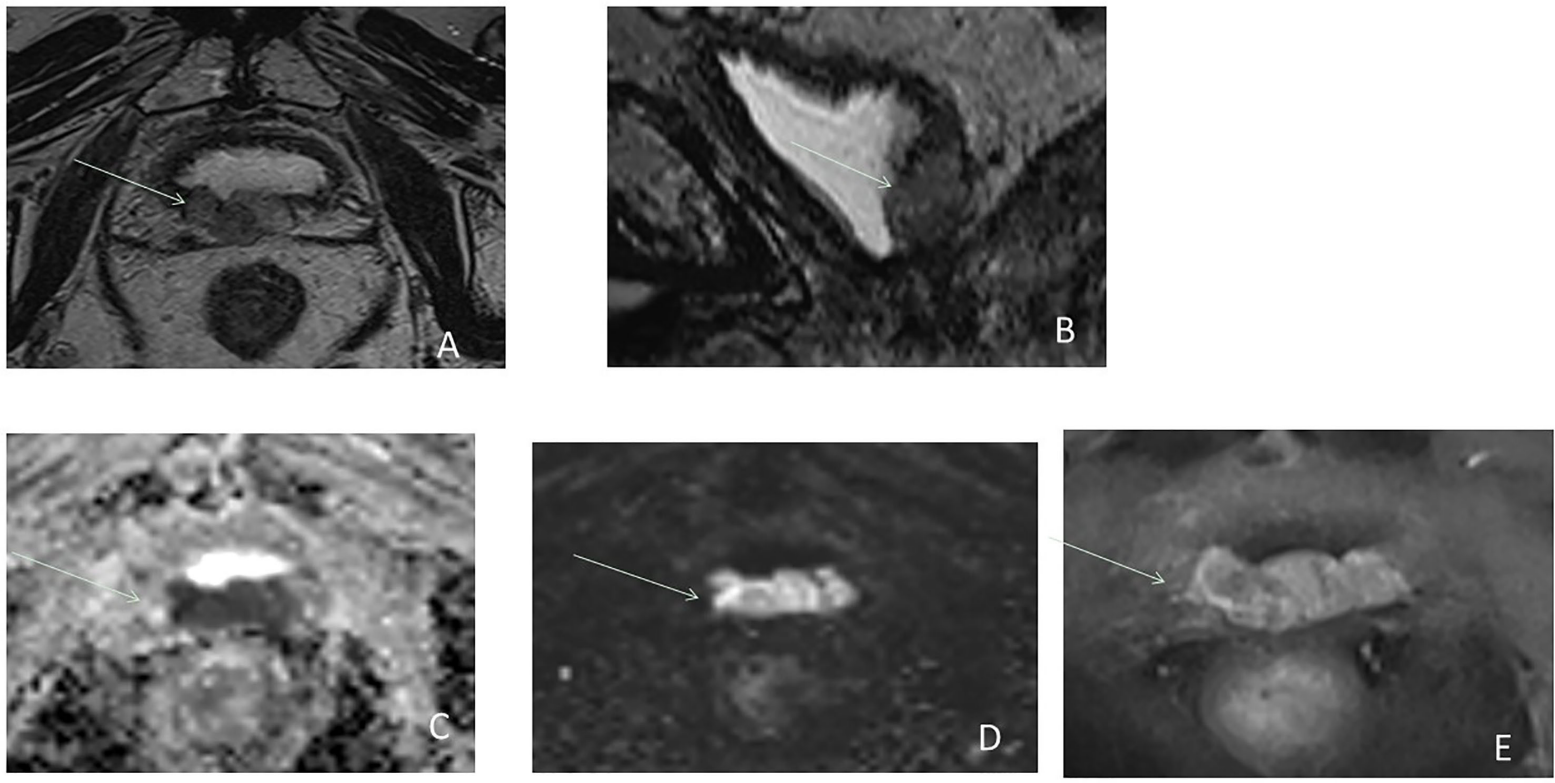
Postsurgical lesion recurrence (arrow) in a 63-year-old male with serum PSA = 0.8 ng/ml. Axial T2W MRI **(A)**, sagittal T2W **(B)**, ADC map **(C)**, b2000 DW MRI **(D)**, and DCE MRI **(E)** show a large lobulated mass relatively hyperintense on T2W, hypointense area on the ADC map, hyperintense area on b2000, and with early hyperenhancement located in the postpart of the bladder neck.

Local recurrence usually presents as a nodular, semicircumferential to ill-defined soft-tissue mass of intermediate T2-weighted signal intensity with associated diffusion restriction and rapid, early enhancement on dynamic contrast-enhanced imaging; signal characteristics are similar to those of the initial tumor (35) (**Figure 2**).

**Figure 3:**
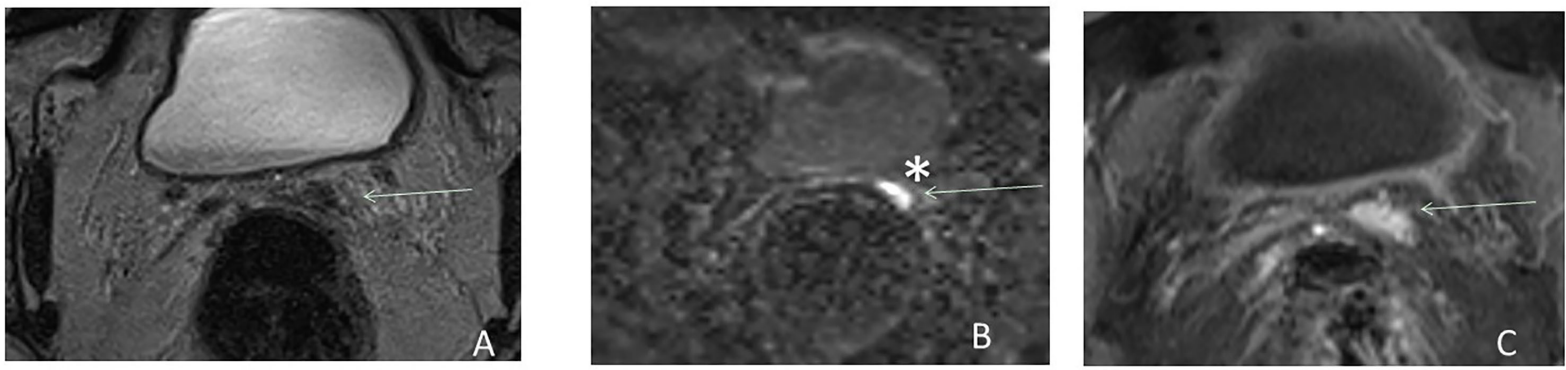
Postsurgical recurrence after RP. Axial T2W MRI **(A)**, b2000 DW MRI **(B)**, and DCE MRI **(C)** show the benefit of functional imaging to detect local recurrence in the left seminal vesicle bed (arrow) and particularly the benefit of high *b*-value ^*^. *Highlight the benefit of high b value.

**Figure 4:**
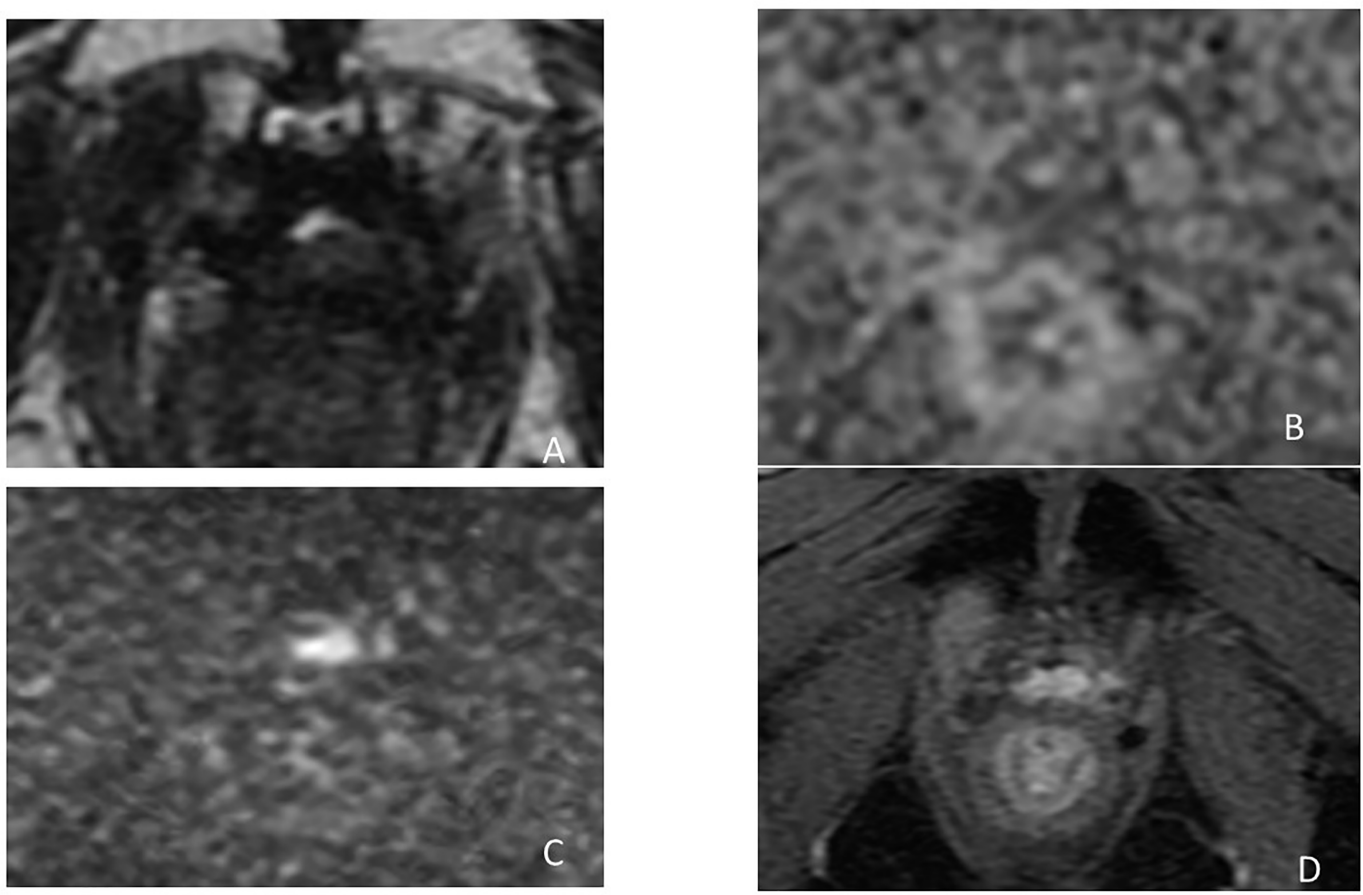
Postsurgical lesion recurrence (arrows) in a 63-year-old male with serum PSA = 0.6 ng/ml after radical prostatectomy. Axial T2W MRI **(A)**, ADC map **(B)**, b2000 DW MRI **(C)**, and DCE MRI **(D)** show a soft tissue lesion at the posterior part of VUA, relatively hyperintense on T2W best seen on high *b*-value and DCE imaging with a focal nodular enhancement that contrasts sharply with the general background low-level venous enhancement.

The value of DW MRI is very variable after RP. Diffusion imaging can be distorted by the presence of surgical clips and susceptibility artifacts. However, DWI can be useful for distinguishing tumor from mimicking etiologies, such as inflammation or residual benign tissue (33) (**Figures 3**, **4**). The performance of diffusion imaging has been evaluated and showed good results, especially with the use of high *b*-values (that reflects the strength and timing of the gradients used to generate diffusion-weighted images). In this specific indication, the values of *b* must be greater than 1,400 s/mm^2^ and up to 3,000 s/mm^2^ (33). In a recent study of 118 patients, Kwon et al. (36) found DW imaging is accurate in distinguishing recurrence from slowly enhancing benign tissue on DCE MRI. Barchetti et al. (37), in a review, concluded its good diagnostic accuracy in detecting local recurrence after RP when combined with other sequences. In a recent systematic review, Sandgren et al. (38) found that DWI combined with T2W imaging had a pool sensitivity of 84% and a pool specificity of 89%. Nevertheless, the addition of DWI was of limited incremental value for detection, especially of small lesions (17). The sensitivity for nodules of sized ≥1 cm is better (39).

DCE is more reliable than DWI and has long been considered the most useful sequence for detecting recurrence. Panebianco et al. (33) found a sensitivity and specificity of 98% and 94% for the detection of a local recurrence for an average PSA of 1.3 ng/ml (0.5–1.7 ng/ml) and an average size of 5 ± 0.6 mm (4 to 8 mm). Similarly, Kitajima et al. (17) found an AUC, sensitivity, and accuracy of mpMRI for detecting local recurrence of 0.91%, 88.5%, and 87.4%, respectively. The authors concluded that the addition of DCE to T2-weighted imaging improves the accuracy of detection of local cancer recurrence after RP and was significantly better than that of PET/CT with choline. De Visschere et al. (40) highlighted that even tiny recurrence “foci” that may not be visible on T2-weighted imaging tends to show a significant enhancement in the early arterial phase, often with contrast washout. Lastly, post-RP recurrences enhance sooner and faster than normal postoperative changes, allowing to distinguish from fibrotic or granulation tissue. Two meta-analyses evaluated the performance of mpMRI for the detection of local recurrence after RP and showed, for DCE combined with T2-weighted imaging, a pooled sensitivities and specificities of 85% and 95% (6) and 84% and 92%, respectively (38).

## Performance of Anastomotic Biopsy After RP

No optimal TRUS-guided biopsy strategy has been defined. Even with TRUS guidance, the sensitivity of anastomotic biopsies is low 40%–71% for PSA levels >1 ng/ml and 14%–45% for PSA levels <1 ng/ml (41, 42).

## Combination of MRI and PET-CT for Detection of Local Recurrence

The use of choline or PSMA PET-CT may help to identify regional or distant recurrences. PSMA PET-CT provides superior accuracy to the conventional imaging CT and bone scanning and is now proposed as a suitable replacement to the conventional imaging (5, 16). However, LR with close proximity and/or infiltration of the bladder is at risk to be missed in 68Ga-PSMA-11-PET as the radioactivity in the bladder may obliterate the visualization of the recurrence (43). The addition of MRI to PET may be useful in the early detection of these occult lesions developing in the bladder neck.

Counago et al. (44), in a retrospective analysis of 38 patients with BCR after RP showed that the combination of both MRI and 18FCH PET-CT gives a better LR detection rate versus choline PET/CT alone. Inferiority for 18F-FCH-PET compared with 68GaPSMA-11-PET was demonstrated for imaging of recurrent PC due to the excellent diagnostic accuracy of 68Ga-PSMA11 for PC-related tissue (45). This underlying potential benefit of the combination of MRI to PET-CT combining the advantage of MRI in obtaining morpho-functional information of local relapse with those of PET to detect nodal or distant recurrences. The use of PSMA-PET/MRI technologies allows the realization of this staging in a single session (46).

Eiber et al. (47) showed that ^11^C-choline PET/MRI improved the detection rate of local relapse compared with ^11^C-choline PET/CT in a prospective study of 75 patients: mean detection rates were respectively 84.7% versus 77.3%. In a study of 119 patients, the detection rate of ^68^Ga-PSMA-11-PET/MRI improved compared with PET/CT, particularly for LR close to the bladder (43). In a recent meta-analysis, PET/MRI had a high sensitivity rate (80.9%–95% CI, 73.0%–86.9%) in detecting relapse (48).

## Delineation of the Clinical Target Volume

Various questions remain unresolved with regard to optimal target volume definition and RT doses that can vary depending on whether the recurrence is micro- or macroscopic (24).

**Figure 5:**
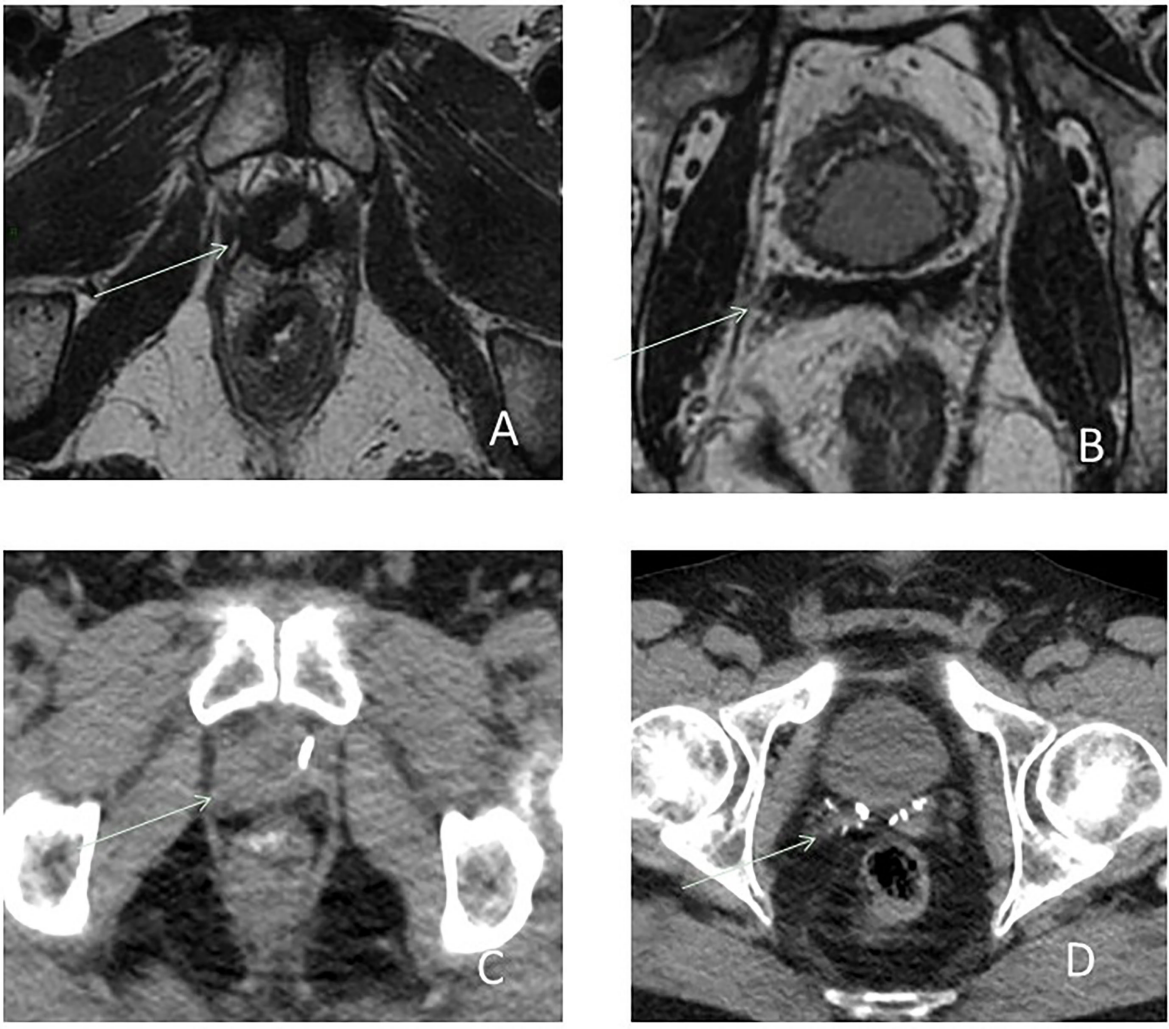
Biological recurrence in a 58-year-old male. Axial T2-weighted images **(A, B)** and CT **(C, D)** showing the benefit of MRI to enable a better delineation of anatomical analysis of the VUA **(A**, **C)**, cut end of the vas deferens and SV bed **(B**, **D)**, and bladder neck, all common sites of recurrences.

However, most of the time recurrence remains microscopic and the volume definition of the CTV corresponds to the prostate bed. Nonetheless, even if MRI is negative, all potential recurrence sites listed in **Table 1** must be included in the CTV. Published consensus guidelines are considering CT as reference imaging system to contribute in CTV delineation (29, 49, 50), except for the Princess Margaret Hospital (PMH) (51), which used postoperative MRI. Potential benefit of MRI is to enable a precise anatomical analysis of the prostate bed, cut end of the vas deferens, SV bed (SV remnants or fibrotic), and bladder neck, all common sites of recurrences (**Figure 5**).

Croke et al. (52) showed that current CTV consensus definition does not adequately cover prostate bed, and that application of the current CTV guidelines results in a localization error in approximately one-third of the target volume base on preoperative MRI-defined prostate volumes.

MRI could transform an invisible target in a visible target volume (18). In the case of an identified recurrence, modification of the CTV could be necessary to avoid underdosage of the recurrence area (53). Image registration between mpMRI and planning CT scan helps to identify accurately the target and decreases the CTV and the doses delivered to organs at risk (54). Moreover, it allows intensification of radiotherapy: Zilli et al. (55) treated 131 patients with a dose of 64 Gy on a surgical bed and added a 10-Gy boost on the recurrence site. Toxicity was not increased and local relapse-free survival was 100%. A concomitant integrated boost is also feasible without increasing toxicity (56). However, the benefit of this boost dose was not demonstrated (55–57).

Identification of a local recurrence on MRI is associated with a better efficacy of SRT. So, negative MRI is an argument to the use of androgen deprivation therapy (ADT), in combination with SRT, particularly if the PSA is higher than 0.5 ng/ml (32, 34, 58).

## Conclusion

Multiparametric MRI could be a useful tool before SRT, showing local or regional relapse with sensitivity and specificity reaching 90%. Combined with PSMA PET-CT, ideally during the same session, it allows to spare some patients with distant relapse from a futile SRT. In the future, it could be proposed to patients with BCR, particularly when the PSA is higher than 0.3–0.5 ng/ml, to facilitate the delineation of target volume and allow dose escalation on a specific area.

## Author Contributions

All authors have participated in the conception and drafting of the manuscript under the supervision of RP, IL, and CH. All authors listed have made a substantial, direct, and intellectual contribution to the work and approved it for publication.

## Conflict of Interest

The authors declare that the research was conducted in the absence of any commercial or financial relationships that could be construed as a potential conflict of interest.

## Publisher’s Note

All claims expressed in this article are solely those of the authors and do not necessarily represent those of their affiliated organizations, or those of the publisher, the editors and the reviewers. Any product that may be evaluated in this article, or claim that may be made by its manufacturer, is not guaranteed or endorsed by the publisher.

## Abbreviations

ADC, apparent diffusion coefficient; ADT, androgen deprivation therapy; DCE, dynamic contrast-enhanced; DWI, diffusion-weighted imaging; EAU, European Association of Urology; ISUP, International Society of Urological Pathology; LR, local recurrence; mpMRI, multiparametric magnetic resonance imaging; NPV, negative predictive value; PPV, positive predictive value; RP, radical prostatectomy; SRT, salvage radiation therapy; SV, seminal vesicle; T2W, T2-weighted; VUA, vesico-urethro-anastomis; yo, years old.
